# A comparative study between intravitreal triamcinolone and bevacizumab for macular edema due to central retinal vein occlusion with poor vision

**DOI:** 10.4103/0301-4738.77008

**Published:** 2011

**Authors:** Ji Won Lim, Kyeong-Ik Na

**Affiliations:** Department of Ophthalmology, Hallym University Sacred Heart Hospital, Korea

**Keywords:** Bevacizumab, central retinal vein occlusion, macular edema, triamcinolone

## Abstract

**Aim:**

To compare the effect of intravitreal bevacizumab and triamcinolone in patients with macular edema after central retinal vein occlusion (CRVO), presenting with poor visual acuity.

**Materials and Methods:**

It was a retrospective, comparative case series of 38 consecutive eyes, with macular edema secondary to CRVO, with 20/200 or worse vision, which were treated primarily either with intravitreal bevacizumab (1.25 mg; 24 eyes) or intravitreal triamcinolone (4 mg; 14 eyes). During follow-up, 3.6 ± 0.8 re-injections of bevacizumab and 2.4 ± 0.5 re-injections of triamcinolone were administered (*P* = 0.080). The main outcome measures were the best-corrected visual acuity and the central macular thickness by optical coherence tomography during 12 months of follow-up.

**Results:**

At 12 months, visual acuity (logMAR) was changed from 1.03 ± 0.39 (baseline) to 0.92 ± 0.39 (*P* = 0.374) and the central macular thickness was reduced from a baseline of 713.6 ± 179.3 µm to 310.8 ± 205.2 µm (*P* = 0.000). Neither the bevacizumab nor triamcinolone groups varied significantly in visual acuity and central macular thickness at 1, 3, 6, and 12 months after treatment. Neovascular glaucoma developed in two of the 14 eyes (14%) in the triamcinolone group.

**Conclusion:**

In patients with CRVO and poor vision, intravitreal bevacizumab and intravitreal triamcinolone were associated with a reduction in macular edema; however, neither treatment achieved significant visual acuity improvement by the 12-month follow-up.

Central retinal vein occlusion (CRVO) causes vision loss as a result of macular edema and / or retinal ischemia.[1] The Central Vein Occlusion study[2] failed to demonstrate a statistically significant visual acuity benefit from grid laser photocoagulation, for macular edema. New modalities have been explored in the treatment of macular edema secondary to CRVO, among which, both intravitreal triamcinolone acetonide and intravitreal bevacizumab have shown a marked reduction in macular edema, also accompanied by improvement in visual acuity.[3–9] Both drugs differ in the spectrum of side effects and potentially in the magnitude and duration of their effect. The aim of the current study is to assess and compare the changes in visual acuity and macular thickness on optical coherence tomography (OCT) and to assess the complications of CRVO with poor vision, in patients receiving either triamcinolone or bevacizumab.

## Materials and Methods

This retrospective, comparative study included 38 eyes (38 patients) with CRVO presenting with 20/200 or worse visual acuity, which consecutively underwent intravitreal injection of either triamcinolone (4.0 mg; 14 eyes) or bevacizumab (1.25 mg; 24 eyes) with at least 12 months of follow-up. Triamcinolone was injected between October 2007 and May 2008. After the use of intraocular bevacizumab was approved in the hospital, bevacizumab was injected between June 2008 and September 2009 by one physician. The same drug was used during the whole study period for each eye. The study had Institutional Research Board approval and informed consent was obtained from all patients.

The inclusion criteria were as follows: The patients had to be with perfused CRVO confirmed by fluorescein angiography, with central macular thickness ≥ 250 µm, and baseline visual acuity of 20/200 or worse. Perfused CRVO was defined as lacking evidence of neovascularization in the retina or iris and having no obvious macular ischemia. The exclusion criteria were previous treatment for CRVO, such as intravitreal injection, subtenon injection, or laser photocoagulation, since the time of onset of CRVO, a history of glaucoma, macular edema secondary to other causes, such as age-related macular degeneration and diabetic retinopathy.

At baseline, all the patients underwent a thorough ophthalmological examination, including best-corrected visual acuity measurement with a Snellen chart, applanation tonometry, ophthalmoscopy, fluorescein angiography, and OCT (OCT, Model 3000; Carl Zeiss Meditec Inc., Dublin, CA, USA). Counting fingers and hand movement at 1 m were converted to 1.6 and 1.9. Four mg (0.1 ml) of crystalline triamcinolone acetonide (Triam; Sinpoong Med., Seoul, Korea) or 1.25 mg (0.05 ml) of bevacizumab (Avastin; Genentech, San Francisco, CA, USA / Hoffmann La Roche, Basel, Switzerland) was injected into the vitreous cavity under sterile conditions. After the injection, a topical antibiotic was applied and the patients were monitored for potential injection-related complications. The patients were initially followed up at the first week post-injection, and twice at two-week intervals, and then at routine monthly intervals.

The main outcome measures were visual acuity and central macular thickness on OCT at one, three, six, and twelve months after the initial injection. The complications during follow-up were also noted. Repeated intravitreal injections were carried out when the central macular thickness appeared to be more than 250 µm on OCT. For triamcinolone, the interval between repeat injections was at least three months, and for bevacizumab, repeat injections were performed at intervals of at least six weeks. One eye in the triamcinolone group received a single intravitreal injection. The remaining 37 eyes received multiple injections. During follow-up, the mean number of injections was 3.8 ± 0.8 in the bevacizumab group (range, 3 – 5 times) and 2.4 ± 0.5 in the triamcinolone group (range, 1 – 3 times).

Statistical analysis was performed using a commercially available statistical software package (SPSS for Windows, version 16.0; SPSS, Chicago, IL, USA). Visual acuity was converted into the logarithm of the minimum angle of resolution (logMAR) for statistical calculations. Univariate categorical analysis was performed using the two paired t-test, chi-square test, Mann-Whitney U test, or Fisher exact test, as appropriate. The data were analyzed via repeated-measures analysis of variance with a Bonferroni correction. The level of statistical significance was set at 0.05 (two-sided) in all statistical testing.

## Results

The mean age of the patients was 68.7 ± 14.7 years (range, 48 – 98 years). The mean duration of symptoms was 10.5 ± 3.4 weeks (range, 4 – 20 weeks). The mean baseline visual acuity (logMAR) was 1.03 ± 0.39 (range, 1 – 1.9) and the mean central macular thickness was 713.6 ± 179.3 µm (range, 433 – 1159 µm). At baseline, the groups did not vary significantly with respect to age, gender, duration of symptoms, visual acuity, central macular thickness, and intraocular pressure [Table 1].

**Table 1:** Baseline characteristics of patients with central retinal vein occlusions, with 20/200 or worse vision

Variables (mean ± SD)	Total (n = 38 eyes)	Bevacizumab group (n = 24 eyes)	Triamcinolone group (n = 14 eyes)	*P*-value
Age (years)	68.7 ± 14.7	67.5 ± 16.6	72.6 ± 2.9	0.109*
Gender, Male : Female	20:18	12:06	08:06	0.445†
Duration of symptoms (weeks)	10.5 ± 3.4	10.2 ± 4.2	11.2 ± 5.4	0.785*
Number of injections	3.0 ± 0.6	3.6 ± 0.8	2.4 ± 0.5	0.080*
VA (logMAR)	1.03 ± 0.39	1.04 ± 0.38	1.00 ± 0.44	0.673*
Central retinal thickness at OCT (µm)	713.6 ± 179.3	716.7 ± 199.1	703.0 ± 99.5	0.324*

SD: Standard deviation,

* Wilcoxon two sample test,

† Fisher’s exact test,

VA: Best corrected visual acuity, logMAR: Logarithm of the minimum angle of resolution, OCT: Optical coherence tomography

During follow-up, recurrence of macular edema occurred in two eyes of the bevacizumab group at six and eight months after initial injection, respectively. And the one eye receiving a single injection of the triamcinolone group showed recurrence of macular edema at six months after the initial injection. These eyes received re-injection, according to the aforementioned criteria.

At the 12-month follow-up, visual acuity improved to 0.92 ± 0.39 from 1.03 ± 0.39 (*P* = 0.374). The visual acuity did not change significantly from baseline at any time during the follow-up (*P* = 0.546, 0.476, 0430, at one, three, and six months, respectively). The differences in visual acuity were not statistically significant between the two groups at any time during the follow-up. Four of the 38 eyes (10.5%) gained more than two lines visual acuity, and 34 of 38 eyes (90%) showed stable visual acuity. At 12 months, three of the 24 eyes (13%) from the bevacizumab group and one of the 14 eyes (7%) from the triamcinolone group improved in the best-corrected visual acuity, more than two lines. The distributions of visual acuity improvement were not different between the two groups (*P* = 0.446). Five of the 38 eyes (13%) had improvement to ≥ 20/200 at 12 months.

**Figure 1:**
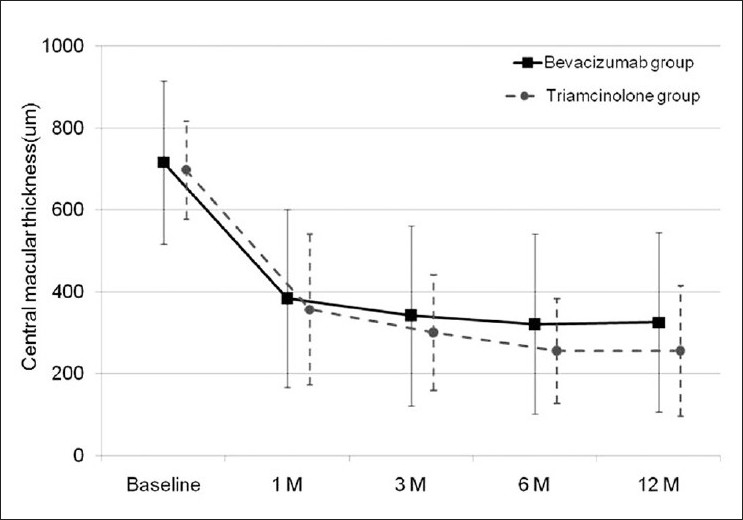
Graph demonstrating changes in the mean central macular thickness (um) after intravitreal bevacizumab and intravitreal triamcinolone injections. Central macular thickness in patients in the intravitreal bevacizumab group (solid line) and in the intravitreal triamcinolone group (dashed line). The bar indicates standard deviation

The central macular thickness was reduced to 310.8 ± 205.2 µm from 713.6 ± 179.3 µm at 12 months (*P* > 0.001). The central macular thickness was reduced more in the triamcinolone group than in the bevacizumab group. However, during follow-up, the central macular thickness between the two groups was not significantly different (*P* = 0.843, 0.730, 0.579, 0.550, at one, three, six, and twelve months, respectively) [Fig. 1]. Three of the 14 eyes (21%) in the triamcinolone group and six of the 24 eyes (25%) in the bevacizumab group had persistent macular edema at 12 months in spite of the repetition of intravitreal injection. The rate of persistent macular edema was not different between the two groups (*P* = 0.454).

In the triamcinolone group, four of the 14 eyes (29%) had intraocular pressures > 21 mmHg without iris rubeosis and a need for topical glaucoma medications. Although, in the bevacizumab group there were no eyes with elevated intraocular pressure after injection, the rate of ocular hypertension between the two groups was different (*P* = 0.050). Rapid progression of cataracts occurred in one of the 14 eyes (7%) in the triamcinolone group. There were no other complications, including vitreous hemorrhage, retinal detachment, and endophthalmitis in the study group. Panretinal photocoagulation was performed on three of the 14 eyes (21%) in the triamcinolone group and on one of the 24 eyes (4%) in the bevacizumab group during the follow-up, to prevent neovascular sequelae (*P* = 0.094).

Two of the 14 eyes (14%) in the triamcinolone group developed iris rubeosis with neovascular glaucoma three and six months from the baseline, respectively. No eyes in the bevacizumab group developed neovascular glaucoma. The rate of neovascular glaucoma between the two groups was not significantly different (*P* = 0.057).

## Discussion

Triamcinolone has been shown to inhibit the expression of the vascular endothelial growth factor (VEGF), in addition to anti-inflammatory properties, anti-permeability function, and neuroprotective effects; Anti-VEGF agents, such as bevacizumab, exert direct, strong inhibition of VEGF.[10] Several studies involving a reduction in macular edema due to CRVO with triamcinolone or bevacizumab have revealed promising results.[11–14] However, the series in which macular edema due to CRVO with 20/200 or worse visual acuity was studied, it has analyzed fewer samples. In our study, there was no difference in visual acuity and central macular thickness between the two groups during follow-up. Therefore, the overall results suggest that both intravitreal bevacizumab and intravitreal triamcinolone are associated with the anatomic resolution of macular edema. However, although there was a definite decrease in macular edema on OCT, this did not translate to a direct visual improvement.

The two major side effects of intravitreal triamcinolone are a steroid-induced increase in intraocular pressure and development of cataracts. In contrast, intravitreal bevacizumab has been seen to be free of complications. The side effects in the triamcinolone group were significantly higher than those in the bevacizumab group in the current study. Thus, in view of the potential complications of intravitreal triamcinolone, bevacizumab may be preferred over triamcinolone, for intravitreal use in CRVO.

An important concern for CRVO is prevention of neovascularization and ischemia and its prompt treatment. The risk of neovasculization increases in eyes with poor visual acuity. It is reported that neovasculization of the iris or angle develops in 44% of the eyes with poor visual acuity during a three-year follow-up.[15] Two of the 38 eyes (5%) developed neovascular glaucoma within 12 months in the current study, which is a lower frequency than the known natural course. However, we have not concluded that the intravitreal drugs are superior in preventing neovascularization, compared to the natural course. The intravitreal drug will be washed out with time and neovascularization can be prevented only by eliminating areas of ischemia by laser ablation, on a permanent basis. Although intravitreal drugs could block the cytokine related to angiogenesis temporarily, the latter raises questions about whether intravitreal bevacizumab or triamcinolone injections are capable of preventing ischemia of CRVO during long-term follow-ups.

This study had the limitation of being a retrospective study based on a small number of patients. Additionally, we did not evaluate the long-term effect after a one-year follow-up. A further study is necessary to elucidate an optimized and effective use for CRVO with macular edema. Also, it will be interesting to elucidate the role of the intravitreal injection, and specifically to check whether it is possible for intravitreal treatment to really reduce the rate of progression to ischemia in CRVO and prevent neovascular glaucoma.

Overall, intravitreal injections of bevacizumab or triamcinolone have shown a decrease in macular edema in CRVO patients with poor visual acuity. However, the results in visual acuity improvement are not promising.
